# Factors and pathways influencing fertility anxiety in reproductive-age male cancer patients: a stress process theory perspective

**DOI:** 10.1530/RAF-25-0099

**Published:** 2026-02-10

**Authors:** Zhaoxia Tian, Xia Ren, Wenke Guo, Xiaolong Du, Hongmei Li, Xiwang Hao, Meilian Zhang, Jun Zhang, Ruishan Sheng

**Affiliations:** ^1^ Fenyang College of Shanxi Medical University, Fenyang City, Shanxi Province, China; ^2^ Fenyang Hospital of Shanxi Province, Fenyang City, Lvliang, Shanxi Province, China

**Keywords:** reproductive-age male cancer patients, fertility anxiety, factors and pathways

## Abstract

**Graphical Abstract:**

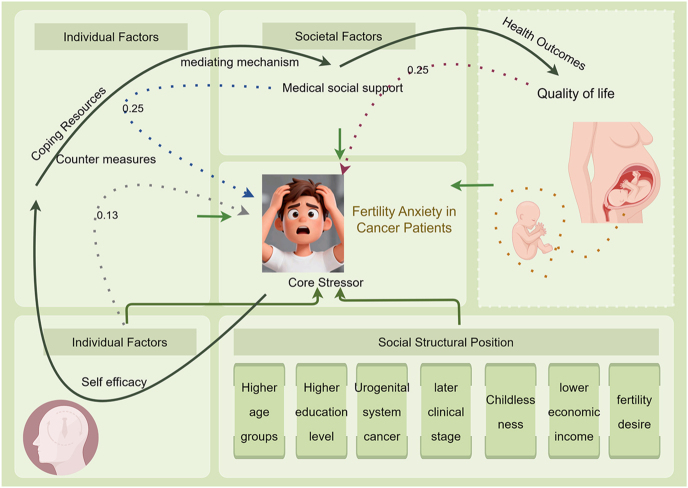

**Abstract:**

**Background**

Fertility concerns among cancer patients of reproductive age are widespread and persist for 5–10 years from diagnosis through treatment, with effects that often surpass those associated with the cancer itself. Unlike female patients who exhibit stronger self-disclosure tendencies, male cancer patients tend to internalize emotions when confronting fertility issues, resisting open discussion or expression of their feelings. Consequently, they experience higher levels of uncertainty and anxiety regarding their fertility status.

**Objective**

To examine the effects and pathways of various variables on fertility concerns among male cancer patients of reproductive age based on the stress process theory.

**Methods**

A multicenter study in Shanxi Province randomly recruited 365 male cancer patients of reproductive age, who completed six questionnaires.

**Results**

Univariate analysis revealed that higher age groups, higher education level, urogenital system cancer, later clinical stage, childlessness, lower economic income, and fertility desire (59.38, 39.599, 8.407, 8.304, 4.877, 9.968, 5.780, *P* < 0.001) were all associated with increased levels of fertility anxiety. Pearson correlation analysis revealed that high quality of life, medical social support, coping strategies, and high self-efficacy (*P* < 0.01) alleviated fertility anxiety, while avoidance and surrender coping strategies increased it. The structural equation model, under the assumed causal order, showed that both medical social support and coping styles exhibited significant indirect effects across all path relationships, constituting partial mediation.

**Conclusion**

The stress process theory reveals diverse influencing variables and pathways for fertility anxiety among male cancer patients of reproductive age. Clinical practice should adopt multidimensional interventions to alleviate anxiety related to fertility issues, enhancing treatment outcomes and achieving therapeutic goals.

**Lay summary**

Cancer can leave young men worried about whether they can ever become fathers. We asked 365 male patients in Shanxi what shaped these worries. Older, less-educated men with advanced or certain cancer types felt more fear, while those who already had children, earned more money, or still wanted children felt calmer. Good social support, better coping skills, and strong self-belief lowered fear, whereas avoidance or giving up made it worse. Doctors should offer emotional, social, and practical help to ease these concerns.

## Introduction

The earlier onset of cancer (25- to 49-year-olds account for 1/10 of cases) (Okyere *et al.* 2024) coupled with the delayed global average age of childbearing (28.6 → 30.9 years) (Liao *et al.* 2024) means that individuals in their reproductive years often have unfulfilled reproductive goals when diagnosed with cancer. Meanwhile, advances in disease screening and treatment technologies have enabled over two-thirds of adolescent and young adult (AYA) cancer patients to achieve a 5-year survival rate exceeding 80% (McDonnell *et al.* 2021), making fertility decline the primary long-term toxicity (Klijn *et al.* 2023, Salsman *et al.* 2025). Over 70% of infertile men experience varying degrees of anxiety, with approximately 11% exhibiting severe anxiety and 30% experiencing major depression (Feng *et al.* 2024, Liao & Jun 2024). Fertility concerns have become an urgent issue for male cancer survivors of reproductive age.

The decline in fertility resulting from cancer treatment is a core factor in fertility concerns among male cancer patients of reproductive age (Quinn *et al.* 2011). In addition, the decline in sexual quality of life and conflicts between family and societal roles significantly exacerbate psychological burdens (Songwathana 2001). Recent studies indicate that many young cancer patients worry about their future fertility, since gonadotoxic treatments can impair fertility, such as the testicular toxicity of alkylating chemotherapy drugs to the testicles, and the risk of permanent infertility increases with cumulative dose (Schover 1999). In addition, post-treatment issues, such as sexual dysfunction and decreased sexual satisfaction, directly impact marital relationships and family stability (Kemerer *et al.* 2023). In specific cultural contexts, men experiencing declining fertility may feel a loss of social identity due to their inability to fulfill the ‘father role’, leading to a crisis of self-identity (Sahoo *et al.* 2025). These factors can all trigger varying degrees of anxiety.

The stress process theory reframes fertility anxiety from an emotional response into a core stressor within a structural stress process. It also views stress as a multifactorial, dynamic process. Through the dynamic model of ‘stressor–mediating mechanism–coping resources–health outcomes’, it reveals fertility anxiety as a critical node of health inequality across the ‘biological–psychological–social’ dimensions (Buss *et al.* 2018). This theory posits that whether an individual experiences psychological or physiological stress responses depends on a series of mediating and moderating factors between the stressor and the stress response. Mediating factors encompass both individual and societal dimensions. Individual factors include coping resources and coping strategies. Coping resources represent an individual’s subjective assessment of their capacity to manage external stressors, reflecting self-efficacy. Coping strategies denote behaviors employed to prevent or avoid stressors and their consequences, manifesting as distinct coping approaches. Societal factors mainly involve the complex and multifaceted concept of social support. Meanwhile, this theory emphasizes the pronounced social gradient in reproductive psychology that emerges from inequalities in social structural positions, such as age, educational attainment, and economic status.

**Figure 1:**
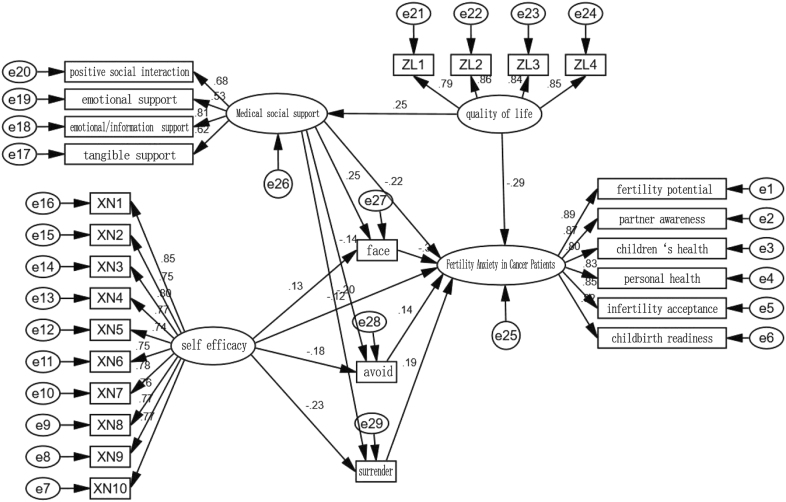
Structural equation model of factors influencing fertility concerns in male cancer patients of reproductive age.

Current research on fertility concerns among male cancer patients of reproductive age has summarized the current state of these concerns and explored assessment tools, potential profiles, influencing factors, and related intervention strategies (Fig. 1). However, several potential determinants and their interaction effects on fertility-related concerns among male cancer patients remain unexplored in current research. Anchored in the stress process theory, this study explores the influence of social structural position on fertility psychology, focusing on exploring the mediating mechanisms linking self-efficacy, social support, and coping modes to fertility-related distress in male oncology patients, in addition to exploring new influencing factors of fertility anxiety and systematically analyzing the pathways of each influencing factor, providing theoretical foundations for developing evidence-based clinical intervention protocols.

**Figure 2:**
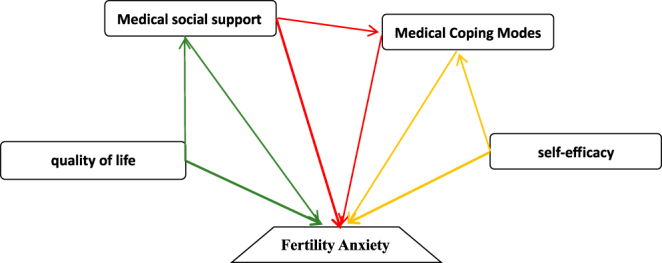
Hypothetical theoretical model (cross-sectional data, unable to verify causal sequence). All paths represent theoretical assumptions rather than causal inferences.

Based on the above research landscape, this paper proposes three theory-driven hypothesis models (Fig. 2).

Hypothesis 1: quality of life indirectly influences fertility concerns among male cancer patients through medical social support, while also potentially exerting a direct effect on fertility concerns. This hypothesis is grounded in the following literature: Hann *et al.* (2025) found a prospective association between quality of life and social support; He *et al.* (2025) observed variations in fertility concerns across different levels of social support.

Hypothesis 2: medical social support indirectly influences fertility anxiety levels through coping modes, while also potentially exerting a direct effect on fertility anxiety. This hypothesis is grounded in the following literature: Martins *et al.* (2016) identified an association between social support and coping strategies; Schmidt (2009) assessed the impact of coping strategies on fertility anxiety.

Hypothesis 3: self-efficacy may indirectly influence fertility anxiety levels through coping modes, while also potentially exerting a direct effect on fertility anxiety. This hypothesis is grounded in the following literature: Zhao *et al.* (2025) revealed a positive association between self-efficacy and coping modes in a risk factor study.

## Subjects and methods

### Subjects

Male patients with malignant tumors meeting inclusion and exclusion criteria were selected from medical data systems across multiple centers in Shanxi Province (total number of individuals contacted: 495; actual participants: 389; participation rate: 78.6%), from October 2022 to October 2024. Inclusion criteria were as follows: i) diagnosed as malignant tumors according to the ‘Diagnosis and Treatment Guidelines for Cancer and Hematological Diseases (2022 Edition)’, ii) age: 22–50 years (based on the World Health Organization (WHO) definition of male reproductive age (15–65 years) and China’s peak fertility age), iii) informed consent and voluntary participation, and iv) clear consciousness and ability to communicate. Exclusion criteria were as follows: i) history of psychiatric or psychological disorders, ii) congenital infertility, and iii) infertility resulting from disorders of the reproductive system. Based on Kendall’s sample size calculation method, the sample size should be 5–10 times the number of independent variables. With 38 variables in this study and accounting for a 20% attrition rate, the required sample size ranged from 228 to 456. The study obtained an effective sample size of 365 cases.

This study was reviewed by the Ethics Committee of the Fenyang Hospital, Shanxi Province (2024038). Patients consent was obtained by an informed consent process that was reviewed by the Ethics Committee of the Fenyang Hospital, which certifies that the study was performed in accordance with the ethical standards laid down in the 1964 Declaration of Helsinki.

### Research tool

#### General information questionnaire

Developed by the research team based on study objectives, this includes age, educational attainment, marital status, children’s status, household income, occupational status, primary caregiver, fertility desire, cancer histology, and clinical stage.

#### Reproductive Concerns After Cancer – Male (RCAC-M)

Developed by Gorman *et al.* (2020), based on the original Reproductive Concerns After Cancer scale, this version specifically measures reproductive concerns among male cancer survivors of reproductive age. The study utilized the Chinese version of the scale translated by Liu *et al.* (2023*a*), which includes six dimensions: fertility potential, partner awareness, children’s health, personal health, infertility acceptance, and childbirth readiness (3 items per dimension), totaling 18 items. Higher scores indicate greater reproductive concerns among patients. The Cronbach’s α coefficient for this scale is 0.827.

#### Medical Outcomes Study Social Support Survey (MOS-SSS)

Revised by Huan Li into the MOS-SSS scale (Li 2012), this scale includes four dimensions with twenty items, meaning that higher scores indicate greater levels of social support. The total scale has a Cronbach’s α coefficient of 0.889.

#### General Self-Efficacy Scale (GSES)

Designed by Schwarze in 1981 and later revised by Wang *et al.* (2001) to include 10 items as a unidimensional scale, it is used to measure an individual’s confidence in overcoming various setbacks or difficulties. A higher score indicates greater self-efficacy in the patient. The Cronbach’s α coefficient for this scale is 0.87–0.90.

#### Medical Coping Modes Questionnaire (MCMQ)

Revised by Shen & Jiang (2000), this tool assesses patients’ coping strategies toward illness. It comprises three dimensions: avoidance, confrontation, and surrender, with a total of 20 items. The Cronbach’s α coefficient for the scale is 0.79.

#### Functional Assessment of Cancer Therapy – Cognitive Function (FACT-Cog)

Developed by Wagner *et al.* (2020), this questionnaire assesses cancer patients’ self-rated cognitive function. The scale consists of 37 items across four subscales. Higher scores indicate less cognitive impairments and better quality of life (QoL). The Cronbach α coefficients for each subscale range from 0.76 to 0.94.

#### Data collection method

After obtaining approval from department heads, the research team conducted in-person surveys in hospital wards. Surveys were administered via paper questionnaires or the online platform WenjuanXing, taking 30–40 min per participant. Patients were informed about the study’s purpose and significance before providing informed consent. A total of 389 questionnaires were distributed. After excluding 24 invalid questionnaires with missing or incorrect entries, 365 valid questionnaires were recovered, yielding a valid response rate of 93.8%.

### Statistical analysis

Data were processed and analyzed using SPSS 26 and AMOS 24. Count data were described using frequencies and percentages. Normality testing is performed on the quantitative data to assess whether the data conform to a normal distribution. Normally distributed data were characterized by mean and standard deviation, while non-normally distributed data were described using median and interquartile range. Inferential statistics included independent-samples *t*-tests, one-way ANOVA, correlation analysis, and exploratory statistics for structural equation models within assumed frameworks. At a 95% confidence level, *P* < 0.05 indicates statistically significant results.

## Results

### Univariate analysis of fertility concerns among male cancer patients of childbearing age

The mean fertility concern score among male cancer patients of reproductive age was 59.02 ± 20.98 points. Univariate analysis revealed significant associations with age (*F* = 59.38, *P* < 0.001), educational attainment (*F* = 39.599, *P* < 0.001), presence of children (*t* = 4.877, *P* < 0.001), per capita monthly household income (*F* = 9.968, *P* < 0.001), fertility desire (*t* = 5.78, *P* < 0.001), cancer clinical stage (*F* = 8.304, *P* < 0.001), and cancer histological type (*F* = 8.407, *P* < 0.001), which were significantly associated with fertility concerns among male cancer patients. Therefore, these factors were selected for inclusion in subsequent multivariate analysis models (Table 1).

**Table 1:** Univariate analysis of fertility concerns in male cancer patients.

Project	*n*	Mean ± SD	*F*/*t*	*P*
Age			59.380	<0.001
20–25 years	120	42.11 ± 17.87		
26–30 years	102	64.25 ± 17.52		
31–35 years	85	68.62 ± 16.99		
35 years and above	58	70.76 ± 15.7		
Ethnicity			0.066	0.947
The Han nationality	315	59.05 ± 21		
Others	50	58.84 ± 21.06		
Education level			39.599	<0.001
Junior high school or below	53	41.51 ± 18.16		
High school (vocational/technical)	68	47.81 ± 16.65		
College	96	61.49 ± 18.61		
Bachelor’s degree or above	148	68.84 ± 18.79		
Marital status			−1.344	0.180
Married	106	56.72 ± 20.17		
Unmarried	259	59.97 ± 21.27		
Children			4.877	<0.001
None	292	61.62 ± 20.67		
With children	73	48.63 ± 19.02		
Per capita monthly household income (¥)			9.968	<0.001
2,000 and below	91	63.02 ± 20.11		
2,001–5,000	136	62.96 ± 19.88		
5,001–8,000	62	60.11 ± 20.12		
8,001–10,000	45	46.62 ± 19.2		
Over 10,000	31	45.81 ± 21.28		
Employment status			−1.130	0.259
Yes	285	58.36 ± 21.17		
No	80	61.36 ± 20.26		
Payment method			0.130	0.985
Provincial medical insurance	59	59.27 ± 19.14		
Municipal medical insurance	52	58.06 ± 20.92		
Resident medical insurance	158	58.77 ± 22.14		
Commercial health insurance	16	59 ± 19		
At one’s own expense	68	60.56 ± 21.03		
Other	12	56.67 ± 19.99		
Fertility desire			5.780	<0.001
Yes	246	63.25 ± 19.31		
No	119	50.28 ± 21.66		
Operative treatment			−0.272	0.785
Yes	89	58.49 ± 21.29		
No	276	59.19 ± 20.92		
Cancer metastasis or recurrence			1.009	0.314
Yes	56	61.63 ± 20.92		
No	309	58.55 ± 20.99		
Cancer clinical staging			8.304	<0.001
Stage I	126	53.94 ± 21.96		
Stage II	124	57.27 ± 21.48		
Stage III	49	64.63 ± 17.67		
Stage IV	66	67.86 ± 16.59		
Cancer tissue type			8.407	<0.001
Epithelial tissue cancer	137	56.64 ± 21.36		
Lymphatic tissue cancer	99	58.18 ± 20.92		
Hematopoietic system cancer	80	57.41 ± 20.91		
Central nervous system cancer	24	58.08 ± 17.04		
Urinary and reproductive system cancer	25	81.48 ± 3.85		
Primary caregiver			0.525	0.592
Spouse	42	57.29 ± 22.59		
Parents	256	58.74 ± 20.96		
Others	67	61.19 ± 20.14		

### Correlation analysis of fertility concerns in male cancer patients of reproductive age

Pearson correlation analysis revealed significant negative correlations between fertility concerns and quality of life (*r* = −0.456, *P* < 0.01), medical social support (*r* = −0.367, *P* < 0.01), coping strategies (*r* = −0.488, *P* < 0.01), and self-efficacy (*r* = −0.321, *P* < 0.01) among cancer patients. Conversely, avoidance and surrender showed significant positive correlations with fertility concerns (*P* < 0.01), and self-efficacy (*r* = −0.321, *P* < 0.01) showed significant negative correlations with fertility concerns among cancer patients. Conversely, avoidance and surrender exhibited positive correlations with fertility concerns, with correlation coefficients of 0.332 and 0.418, respectively (Table 2).

**Table 2:** Correlation analysis of fertility concerns in male cancer patients.

Variable	Cancer patients'	Quality of life	Medical social support	Facing concerns	Avoidance	Surrender	Self-efficacy
	fertility concerns						
Cancer patients worry about having children	1						
Quality of life	−0.456*	1					
Medical social support	−0.367*	0.194*	1				
Facing concerns	−0.488*	0.268*	0.221*	1			
Avoidance	0.332*	−0.196*	−0.135*	−0.168*	1		
Surrender	0.418*	−0.202*	−0.199*	−0.266*	0.321*	1	
Self-efficacy	−0.321*	0.216*	0.214*	0.167*	−0.199*	−0.257*	1
Mean	59.02	8.94	69.04	22.11	16.91	11.67	29.16
SD	20.98	4.87	15.45	6.23	5.83	4.52	7.67

* *P* < 0.01.

### Construction of structural equation model of fertility concerns among male cancer patients of childbearing age

The structural equation modeling constructed through Amos24 was used to examine the impact relationships between factors such as fertility concerns, quality of life, and medical social support in cancer patients. In the model fit assessment, the absolute fit indicator *χ*^2^/df (chi-square-to-degrees of freedom ratio) was 1.487, RMSEA (root mean square error of approximation) was 0.037, GFI (goodness-of-fit index) was 0.915, NFI (normative fit index) was 0.925, TLI (total-less-invariant) was 0.971, and CFI (comparative fit index) was 0.974. The measured results of all indicators are at an excellent level, indicating that the constructed structural equation model demonstrated an excellent model fit (Table 3).

**Table 3:** Model fit test.

Fit indicators	Reference standard	Actual test results
*χ*^2^/d*f*		1.487
Excellent	1–3	
Good	3–5	
RMSEA		0.037
Excellent	<0.05	
Good	<0.08	
GFI	>0.9	0.915
NFI	>0.9	0.925
TLI	>0.9	0.971
CFI	>0.9	0.974

The path analysis results of the structural equation model indicate that all hypothesized paths proposed in this study reached statistical significance (*P* < 0.05). In particular, quality of life (−0.29), medical social support (−0.22), self-efficacy (−0.12), and coping mode (facing) (−0.31) exhibited significant negative relationships with fertility concerns among male cancer patients, suggesting that these factors alleviate such anxieties. Avoidance (0.14) and surrender (0.19) coping strategies can exacerbate fertility concerns among cancer patients (Table 4).

**Table 4:** Path relationship tests in structural equation modeling.

Path relationship			NSC	SC	SE	CR	*P*
Medical social support	←	Quality of Life	0.586	0.253	0.148	3.948	***
Facing concerns	←	Medical social support	0.620	0.250	0.148	4.188	***
Avoidance	←	Medical social support	−0.331	−0.143	0.135	−2.455	0.014
Surrender	←	Medical social support	−0.362	−0.202	0.104	−3.474	***
Facing concerns	←	Self-efficacy	1.103	0.126	0.461	2.391	0.017
Avoidance	←	Self-efficacy	−1.499	−0.183	0.438	−3.423	***
Surrender	←	Self-efficacy	−1.454	−0.229	0.334	−4.346	***
Cancer patients' fertility concerns	←	Facing concerns	−0.156	−0.306	0.023	−6.844	***
Cancer patients' fertility concerns	←	Avoidance	0.074	0.135	0.024	3.117	0.002
Cancer patients' fertility concerns	←	Surrender	0.134	0.190	0.032	4.247	***
Cancer patients' fertility concerns	←	Quality of Life	−0.839	−0.286	0.141	−5.962	***
Cancer patients' fertility concerns	←	Self-efficacy	−0.558	−0.125	0.207	−2.699	0.007
Cancer patients' fertility concerns	←	Medical social support	−0.273	−0.216	0.070	−3.884	***

NSC, non-standardized coefficient; SC, standardized coefficient.

*** *P*<0.001.

This study employed a bootstrapping method with 5,000 iterations to calculate 95% confidence intervals, thereby further validating the mediating effects. Results indicate that medical social support partially mediated 16% of the association between quality of life and fertility concerns among cancer patients (indirect effect = 0.16, *P* < 0.001); coping styles partially mediated 26.2% of the association between social and medical support and fertility concerns among cancer patients (indirect effect = 0.097, *P* < 0.001); and coping modes partially mediated 25.9% of the association between self-efficacy and fertility concerns among cancer patients (indirect effect = 0.195, *P* < 0.001) (Table 5).

**Table 5:** Bootstrap (5,000 iterations) mediation effect tests.

Path relationship/effect type	Effect size	95% CI		*P*	Effect contribution
		LLCI	ULCI		
Quality of life → medical social support → fertility concerns in cancer patients					
Indirect effect	−0.160	−0.301	−0.073	<0.001	16.0%
Direct effect	−0.839	−1.142	−0.529	0.001	84.0%
Total effect	−0.999	−1.308	−0.699	<0.001	100.0%
Medical social support → facing concerns→ fertility concerns in cancer patients					
Indirect effect	−0.097	−0.171	−0.046	<0.001	26.2%
Direct effect	−0.273	−0.440	−0.134	<0.001	73.8%
Total effect	−0.370	−0.550	−0.227	<0.001	100.0%
Medical social support → avoidance → fertility concerns in cancer patients					
Indirect effect	−0.024	−0.067	−0.004	0.013	8.1%
Direct effect	−0.273	−0.440	−0.134	<0.001	91.9%
Total effect	−0.297	−0.468	−0.153	<0.001	100.0%
Medical social support → surrender → fertility concerns in cancer patients					
Indirect effect	−0.049	−0.105	−0.017	0.002	15.3%
Direct effect	−0.273	−0.440	−0.134	<0.001	85.0%
Total effect	−0.321	−0.502	−0.177	<0.001	100.0%
Self-efficacy → avoidance → fertility concerns in cancer patients					
Indirect effect	−0.173	−0.351	−0.016	0.031	23.7%
Direct effect	−0.558	−0.987	−0.133	0.010	76.3%
Total effect	−0.731	−1.162	−0.319	0.001	100.0%
Self-efficacy → surrender → fertility concerns in cancer patients					
Indirect effect	−0.111	−0.254	−0.028	0.006	16.6%
Direct effect	−0.558	−0.987	−0.133	0.010	83.4%
Total effect	−0.669	−1.099	−0.244	0.004	100.0%
Self-efficacy → facing concerns → fertility concerns in cancer patients					
Indirect effect	−0.195	−0.374	−0.087	<0.001	25.9%
Direct effect	−0.558	−0.987	−0.133	0.010	74.1%
Total effect	−0.753	−1.162	−0.339	0.001	100.0%

## Discussion

The peak incidence age for certain tumor-related diseases overlaps with the reproductive years. As cancer treatment regimens improve and survival rates increase, the focus of cancer care has gradually shifted toward long-term effects, including reproductive outcomes and fertility impairment (Taylor *et al.* 2016). A study involving 149 patients with various tumor-related diseases found that more than two-thirds of those who had not completed their fertility plans discussed fertility issues with their oncologists (Geue *et al.* 2014). Cancer-related infertility profoundly impacts the psychosocial functioning of both women and men (Szkodziak *et al.* 2020); the psychological aspects of male infertility are often overlooked and undertreated (Allocca *et al.* 2018). The severity of mental distress in male patients, such as generalized anxiety disorder, major depressive disorder (Chen *et al.* 2022), and sexual dysfunction, will fluctuate with different stages of fertility treatment (Ronchi & Napoletano 2021). Considering the profound psychological effects of declining or lost fertility in cancer survivors, it is crucial to further investigate their psychological needs and emotional support required. Therefore, this study employs a stress-process theoretical model, incorporating variables of quality of life, social support, self-efficacy, and stress coping, to explore the relevant pathways influencing fertility concerns among cancer patients of reproductive age.

### Effects of multiple factors on fertility concerns

Our findings indicate that urogenital cancers (*β* = 0.153), advanced age (*β* = 0.329), and later clinical stages (*β* = 0.155) significantly exacerbate fertility concerns among male cancer patients. This aligns with the conclusions of Goldenberg *et al.* (2025). In this review, reproductive concerns among urogenital cancer patients (81.48 ± 3.85) were higher than those with cancers of other systems, which is consistent with the findings of Ofman (1993). The impact of urogenital cancers on fertility extends beyond treatment-related damage to gonadal function, such as cumulative chemotherapy doses or surgical removal of testes or penis, to include alterations in sex hormone receptor expression and sexual dysfunction syndromes. Simultaneously, during the pre-treatment decision-making phase regarding fertility, emotional needs regarding future fertility are often overlooked, leading to higher levels of anxiety. Cancer patients aged 20–35 exhibit heightened anxiety about future fertility and greater involvement in fertility preservation decisions, aligning with the findings of Adams *et al.* (2013) fertility preservation among cancer survivors. However, this study found that patients with advanced-stage cancer, due to extended survival periods or prolonged chemotherapy affecting the reproductive system, tend to be more concerned about fertility and future childbearing potential; their perception of cancer’s potential threat to fertility is more pronounced (Liu *et al.* 2023*b*). Higher educational attainment (*β* = 0.324) also accelerated fertility concerns in this population, potentially linked to their greater knowledge demands, autonomy, independent thinking abilities, and participation and decision-making control over fertility preservation (Wu *et al.* 2019).

This study found that patients with strong fertility desires (*β* = −0.138) and low economic income (*β* = −0.145) exhibited higher levels of fertility anxiety. Those desiring children showed greater anxiety than those without such desires, worrying about reproductive prospects and the impact of disease and treatment on offspring health outcomes, which is consistent with the findings of Nahata *et al.* (2019); conversely, favorable economic circumstances mitigate fertility concerns. Household income directly influences reproductive intent, while cancer treatment, post-treatment rehabilitation, and pre-treatment reproductive interventions all demand substantial financial resources. Unstable economic foundations heighten patients’ caution and anxiety regarding fertility decisions.

In summary, medical teams should consider the challenges posed by the above factors when serving patients. In addition to technical support, they should effectively identify and assess patients’ psychological state, economic situations, and family circumstances to ensure fertility preservation success rates and alleviate patient anxiety levels.

### Pathways of different factors on fertility concerns

The path analysis results of this study align with theoretical assumptions. Self-efficacy (−0.125), social support (−0.216), and quality of life (−0.286) can independently influence fertility concerns among cancer patients, while also exerting indirect effects through mediating variables. However, due to the cross-sectional design, only path strengths within the hypothesized model can be assessed. Therefore, the mediation analysis in this study should be regarded as exploratory rather than confirmatory.

High self-efficacy can reduce high fertility anxiety levels in male cancer patients, whose abilities in disease comprehension and information processing literacy mitigate anxiety stemming from disease uncertainties. This aligns with the findings from Lingens’ research on social psychological cancer services (Lingens *et al.* 2021). However, the path model hypothesis suggests that self-efficacy may also exert an indirect effect on reproductive concerns through positive coping modes as a mediator (*β* = 0.126). Patients with higher self-efficacy are more likely to adopt proactive coping strategies, such as seeking social support and utilizing psychological intervention resources to mitigate fertility concerns.

Social support, particularly familial social support and medical information support, serves as a potent protective factor against anxiety, aligning with the findings of Shafierizi *et al.* (2022). Patients with lower quality of life may experience heightened anxiety when abandoning reproductive referrals for various reasons. Clinically, understanding patients’ quality of life can predict their willingness to abandon or continue treatment while identifying those at a higher risk of greater psychological distress (Braverman *et al.* 2024). This is consistent with the findings from Quinn *et al.* (2015). However, this study indicates that under the theoretical pathway, social support can also act as a partial mediator (0.253) to improve patients’ quality of life, thereby reducing anxiety levels. Optimizing support resources, such as treatment plans from professional reproductive therapy teams and emotional support from friends, partners, and parents, can improve patients’ quality of life, increase optimism, and consequently lower anxiety stemming from cancer-related fertility issues. In addition, the theoretical pathway of this study posits that coping modes (0.250) play a partial mediating role between social support and fertility concerns. Cancer patients with sufficient social support are more likely to adopt proactive and effective coping measures, such as skillfully seeking beneficial resources and emotional values from family, friends, and other relationships. This protective effect during fertility decision-making before cancer treatment helps reduce their anxiety levels; this aligns with the findings from Wang *et al.*’s (2021) study on breast cancer patients of childbearing age. However, it contradicts conclusions from Qian *et al.*’s (2024) research on thyroid cancer patients, which may be related to the selection of cancer types. Further investigation into fertility concerns among young thyroid cancer patients is warranted.

Overall, fertility concerns among male cancer patients of reproductive age involve multifaceted, complexly interrelated factors with significant partial mediation effects. This offers a new perspective for clinical team. Based on patients’ baseline conditions, identifying their fertility-related psychological stressors enables more precise assessment of core variables affecting individual reproductive psychological adaptation during diagnosis and treatment. This facilitates targeted psychological counseling to help patients access and utilize necessary resources. Concurrently, establishing a structured fertility support system, centered on hospital oncology departments and reproductive centers, with community fertility support stations as hubs, can serve every cancer family with reproductive needs. Furthermore, this theory can guide the development of clinical intervention protocols, promote ongoing multidisciplinary discussions among healthcare providers, enhance their understanding of patients’ psychological needs, and facilitate the creation of strategies to support fertility-related cancer issues while establishing accessible care systems. In clinical practice, when an independent intervention factor fails to achieve the expected anxiety-reduction effect, incorporating relevant mediating factors can enrich the intervention plan. Monitoring these interventions and reinforcing their effects aims to achieve optimal therapeutic outcomes.

## Conclusion

This study is grounded in stress process theory and identifies key factors influencing fertility issues among male cancer patients of reproductive age, including sociodemographics (older age, higher education, urogenital cancers, later clinical stages, childlessness, lower income, and fertility desire), self-efficacy, social support, and quality of life. These factors operate through three primary pathways under the hypothetical model: self-efficacy alters coping strategies, thereby increasing fertility awareness; social support facilitates coping strategies, thereby improving fertility outcomes; and quality of life enhances social support, consequently alleviating fertility concerns. This finding reveals exploratory factors regarding the gap between fertility preservation needs, services, and policies for male cancer patients. It can inform national healthcare policies and medical insurance systems during priority funding reviews, placing ‘cancer fertility preservation’ on the ‘reserve list’ for medical insurance negotiations. It encourages positioning male cancer fertility support as an emerging target for public health research, simultaneously providing a theoretical foundation for piloting small-scale feasibility studies of a tripartite fertility support network involving hospitals, communities, and families.

### Limitation

This study employs a cross-sectional questionnaire approach to explore sensitive topics related to cancer fertility, which may introduce social desirability effects or recall bias and cannot establish definitive causal relationships between variables. Therefore, the mediating pathway should be regarded as an exploratory finding. Future research should employ longitudinal or experimental designs to further validate the causality of this pathway. Qualitative research may also be integrated to supplement deeper insights that questionnaires cannot capture.

## Declaration of interest

All authors state that they have no known competing financial interests or personal ties that may have seemed to affect the work presented in this publication; hence, no possible conflicts of interest are required to be disclosed.

## Funding

This work was supported by Science and Technology Project of Lvliang City, Key Laboratory Project of Clinical Nursing Research (No. 2020ZDSYS15), and Research Project of Shanxi Provincial Administration of Traditional Chinese Medicine (No. 2024ZYYB080).

## Author contribution statement

ZT and XR took part in drafting, revising, analyzing, and discussing the data. Others participated in issuing the scale and collecting and sorting the data. HL gave final approval of the version to be published, agreeing on the journal to which the article has been submitted and agreeing to be accountable for all aspects of the work. Other authors made a contribution to acquiring data.
